# Sense of Community and Quality of Life for Older Adults In Puerto Rico 2 Years After Hurricane Maria

**DOI:** 10.1093/geroni/igaa057.074

**Published:** 2020-12-16

**Authors:** Tommy Buckley, Denise Burnette, Humberto Fabelo, Mauricio Yabar

**Affiliations:** Virginia Commonwealth University, Richmond, Virginia, United States

## Abstract

Psychological sense of community plays an important role in the wellbeing of older adults, especially in cultures that revolve around community involvement and support. In 2017, Hurricane Maria devastated Puerto Rico, deeply altering community structure and social life. This study draws on the Ecological Theory of Aging to test the proposition that older adults’ sense of community is associated with self-assessed quality of life two years later. We conducted face-to-face interviews with a non-probability sample of 154 community-dwelling adults aged 60+ in Puerto Rico. We measured sense of community with the Brief Sense of Community Scale (BSCS) (range 0-32, mean= 24.75, SD= 6.04) and quality of life (QOL) with the EUROHIS-QOL 8-item index (range 0-32, mean= 21.61, SD=5.92). We used multiple linear regression to test the association of sense of community and quality of life while controlling for relevant covariates. Higher levels of sense of community were associated with better quality of life (β=0.270, p<0.001), while increased mental health symptoms (β=-0.557, p<0.001) and poor self-rated health (β=2.964, SE=7.17, p<0.001) were associated with lower quality of life. Findings indicate that sense of community is an important contributor to older adult’s quality of life in Puerto Rico, perhaps in part due to cultural and age-related factors. Moreover, sense of community may serve as a protective factor against adverse outcomes after large scale natural disasters. Researchers should continue to examine this association while advocating for and developing policies and programs that promote older adults' sense of community in post-disaster contexts.

